# Leadership skills perceptions in training – the EFPT perspective

**DOI:** 10.1192/j.eurpsy.2023.205

**Published:** 2023-07-19

**Authors:** M. J. Santos

**Affiliations:** Mental Health Department, Hospital Prof. Doutor Fernando Fonseca, Amadora, Portugal

## Abstract

**Abstract:**

Leadership in healthcare organisations is crucial to continually improve and provide adequate and quality care. Leadership development and training empower psychiatrists to develop leadership skills. Focusing on how to enhance leadership development through leadership skills training and experiential learning should be a priority.

Nonetheless, there is a perception that, throughout Europe, the acquisition of basic or more advanced leadership skills is very seldom part of the professional training.

In 2019, a Leadership Working Group (LWG) was set up within the European Federation of Psychiatric Trainees (EFPT). Its aim is to provide trainees an opportunity to develop their leadership skills.

This presentation will show case some of the efforts by the LWG. A special focus will be given to a European-based cross-sectional survey of needs about leadership skills training in Psychiatry aimed at ECPs, that showed that a majority of ECPs had no access to leadership skills training within curricula and that all the respondents requested it to be included in a psychiatric training program in either optional or mandatory fashion.

**Disclosure of Interest:**

None Declared

